# Combustion Characteristics and Emissions of 99% Cracked Ammonia Blends in a Gas Turbine Representative Swirl

**DOI:** 10.1021/acs.energyfuels.5c05963

**Published:** 2026-02-10

**Authors:** Mustafa Alnaeli, Luca Mazzotta, Rachele Lamioni, Steven Morris, Agustin Valera-Medina

**Affiliations:** † College of Physical Sciences and Engineering, 2112 Cardiff University, Queen’s Building, CF24 3AA Cardiff, U.K.; ‡ Department of Mechanical and Aerospace Engineering, 9311 Sapienza University of Rome, Via Eudossiana 18, 00185 Rome, Italy; § Department of Industrial and Civil Engineering, University of Pisa, L.go L. Lazzarino 2, 56126 Pisa, Italy

## Abstract

Ammonia is increasingly seen as a promising fuel for combustion applications, including gas turbines, due to its hydrogen content and ease of storage, making it a potential method for storing renewable energy. However, using ammonia directly poses challenges in controlling NO_*x*_ emissions, especially for retrofitting existing gas turbines. Cracking ammonia to produce hydrogen and nitrogen could mitigate this issue, although uncracked ammonia traces may remain due to inefficiencies. Therefore, this paper evaluates the impacts on swirling flames, representative of gas turbine combustors, when highly cracked ammonia (17.5% H_2_, 1.0% NH_3_, and 81.5% N_2_) is used. Experiments were conducted at pressures ranging from 1.1 to 6 bar absolute, with air preheated to 500 K and a constant power output of 22.7 kW maintained under lean conditions (equivalence ratio ∼ 0.545) throughout the tests. NH_2_ chemiluminescence intensity increased monotonically with pressure from 1.1 to 6 bar, with a peak intensity observed at 6 bar due to enhanced radical formation at higher collision frequencies. NO_*x*_ emissions rose from 90 ppmv at 1.1 bar to 189 ppmv at 4 bar before stabilizing, indicating a balance between thermal NO_*x*_ formation and ammonia-mediated reduction pathways at higher pressures. NH* intensity decreased with increasing pressure, while OH* radicals remained relatively constant, providing insights into flame structure and reaction zone characteristics. A chemical reactor network model complemented the experimental findings, capturing flame zone dynamics and revealing consistent NO formation pathways through NH_3_, NH_2_, and NNH dissociation across different pressures. These findings demonstrate that pressure significantly influences radical distribution and NO_*x*_ formation mechanisms in highly cracked ammonia combustion, with implications for gas turbine combustor design and emission control strategies. To the authors’ knowledge, this is the first systematic study of pressure-dependent chemiluminescence behavior in highly cracked ammonia swirl flames, providing critical insights for the development of low-emission gas turbine combustors using ammonia-derived fuels.

## Graphical abstract

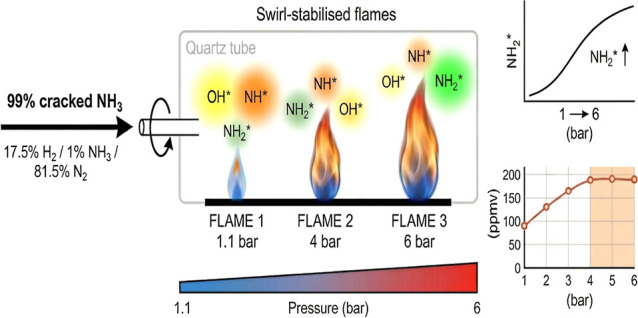

## Introduction

Researchers are actively investigating carbon energy sources to address the increasing global climate challenges. Ammonia (NH_3_) has gained attention as an option, due to its energy density and established production facilities that result in zero carbon emissions when burned.^1–5^ This makes ammonia a compelling solution for reducing carbon footprints across industries, such as power generation.^3,6^ The power industry plays a role in releasing CO_2_ into the atmosphere and could see significant advantages from the potential of ammonia. It can be burned directly in gas turbines and internal combustion engines or used as a carrier for hydrogen fuel cells.^7–11^ Its dual purpose as both fuel and energy storage is valuable for managing intermittent renewable energy, addressing grid stability and energy security challenges.^1,3,12^

Ammonia is being studied in various setups within combustion systems, including cofiring with traditional fuels and utilizing cracked ammonia (hydrogen and nitrogen resulting from thermal breakdown).^4,13–15^ For example, Mitsubishi Power has successfully tested ammonia with coal and burning pure ammonia, achieving steady flames with NO_*x*_ emissions and meeting the desired standards.^9^ This progress reflects the growing interest in ammonia as a utility-scale fuel.

While ammonia presents significant advantages as a carbon-free fuel, including high energy density, established production infrastructure, and suitability as a renewable energy carrier, its application in combustion systems faces several technical challenges. Ammonia’s high hydrogen content makes it a practical hydrogen carrier for the emerging hydrogen economy, and its ease of storage and transportation compared to hydrogen gas enables compatibility with existing power generation infrastructure through retrofitting.^16,17^ However, these advantages must be balanced against significant challenges including high NO_*x*_ formation tendency due to nitrogen content, flame instability and flashback risk (particularly at high hydrogen concentrations), ammonia slip (unburned ammonia emissions that are toxic and environmentally problematic), lower flame speed compared to hydrocarbon fuels requiring specialized burner designs, and material compatibility issues such as ammonia corrosion and hydrogen embrittlement in high-temperature components.^18–20^ Cracked ammonia, which produces hydrogen and nitrogen through thermal decomposition, offers a potential pathway to mitigate some of these challenges by reducing the ammonia content while maintaining energy density. However, the residual ammonia in cracked ammonia fuel (typically 1–5% depending on cracking efficiency) must still be carefully managed to control NO_*x*_ emissions and prevent combustion instabilities.^15,21^

Understanding ammonia’s combustion properties is vital for enhancing efficiency and addressing NO_*x*_ emissions and flame stability. Chemiluminescence has emerged as a method to study these properties by observing emitted light from combustion molecules without interference.^22–24^ Researchers focus on radicals such as NH_2_^*^, NH*, and OH* to gain insights into combustion dynamics. Recent studies have linked chemiluminescence emissions to these radicals, providing valuable information about the flame conditions. Weng et al. conducted a study linking visible chemiluminescence emissions from premixed ammonia–air–oxygen flames to NH_2_^*^ and NO_2_^*^ radicals, showing that intensity ratios can infer equivalence ratios and provide flame condition insights.^12^ Similarly, Karan et al. studied NH_2_^*^ chemiluminescence in shock tube and Bunsen burner setups, concluding that NH_2_^*^ and NH* are strong indicators of maximum heat release rate positions.^25^

Monitoring of radicals NH_2_^*^, NH*, and OH* is essential during ammonia combustion, as they indicate reaction zones and heat release patterns.^25^ While NH* is less intense, it complements the understanding of the flame structure. OH* radicals offer insights into reactivity and temperature distribution within the flame.^12^ Together, these species enhance our understanding of combustion efficiency and emission formation.

Furthermore, examining these radicals through chemiluminescence reveals their impact on combustion dynamics. The arrangement of these radicals highlights regions of temperature and chemical reactivity, which are crucial for assessing material degradation in combustors. Understanding these impacts is critical in the process of crafting and picking materials for power generation systems based on ammonia, hence ensuring the durability and dependability of these infrastructures. This is particularly important for ammonia/hydrogen gas turbines, where high-temperature materials for complex components face challenges such as ammonia corrosion, hydrogen embrittlement, and stress corrosion cracking.^18^

Given these considerations, this study focuses on the combustion characteristics of 99% cracked ammonia, a fuel composition that retains some of the ammonia’s unique properties (i.e., smell). 99% cracked ammonia refers to ammonia that has been thermally decomposed with 99% conversion efficiency through the endothermic reaction 2NH_3_ → N_2_ + 3H_2_, resulting in a fuel composition of 17.5% H_2_, 1.0% NH_3_ (uncracked), and 81.5% N_2_ by volume. Furthermore, it is believed that future cracking systems will not be 100% efficient, hence leaving some traces of NH_3_ to be considered. By investigating the chemiluminescence of NH_2_^*^, NH*, and OH* radicals under various pressure conditions, this study aims to enhance knowledge of ammonia/hydrogen combustion processes. Our findings will not just deepen our understanding of the processes involved in combustion but also offer valuable knowledge for effectively incorporating ammonia into power generation systems by considering factors such as material selection and system design.

## Materials and Methods

Experimental tests are conducted in a High-Pressure Optical Combustor (HPOC), Figure 1, which enables nonadiabatic conditions in the system, which are known to produce higher NO_*x*_ and lower NH_3_ as a consequence of higher reactivity.^26^ Tests were conducted using 99% cracked ammonia blends with air preheated to 500 K, and elevated pressures (from atmospheric to 6 bar absolute). Experiments were carried out at a constant power output of 22.7 kW under lean conditions, as summarized in Table 1. Combustor exhaust gas emissions were sampled downstream of the quartz confinement using a 9-hole equal-area probe, water-conditioned with a heat exchanger to regulate sample temperature (433 K) following specifications in ISO-11042 (British Standard, 1996). Nitric oxide concentrations are quantified using heated vacuum chemiluminescence (Signal 4000VM). Unburned NH_3_ measurements are obtained by redirecting the sample through an NO converter (Signal 410) to measure unreacted concentrations (80% conversion efficiency). All NH_3_ and NO concentrations are measured hot/wet and normalized to equivalent dry conditions (ISO-11042). Dry O_2_ concentrations are quantified using a paramagnetic analyzer (Signal 9000MGA) and used to subsequently normalize NO to equivalent 15% O_2_ (ISO-11042). The total uncertainty of the measurements was determined to be less than 5%, calculated using the Root Sum of Squares (RSS) method. This comprehensive approach accounts for multiple sources of uncertainty, including analyzer specifications, linearization, and span gas certification. To ensure data robustness and account for random fluctuations, each experimental condition was repeated, yielding over 60 data points for each measurement. The standard deviation of these repeated measurements is represented by error bars in Figures 4 and 5, providing a clear visual indication of the data’s stability and consistency. A pair of LaVision CCD cameras was employed to visualize OH*, NH*, and NH_2_^*^ at a frequency of 10 Hz and a gain of 85%. LaVision Davis v10 was used to gather 200 frames per flame, which were then postprocessed using a MATLAB script designed to conduct Abel deconvolution averaging. Averaged calibration data was used to determine the radius of the image based on a separation of 10 mm between holes, and a color map of each image was produced to determine the location of the central pixel column.

**Figure 1:**
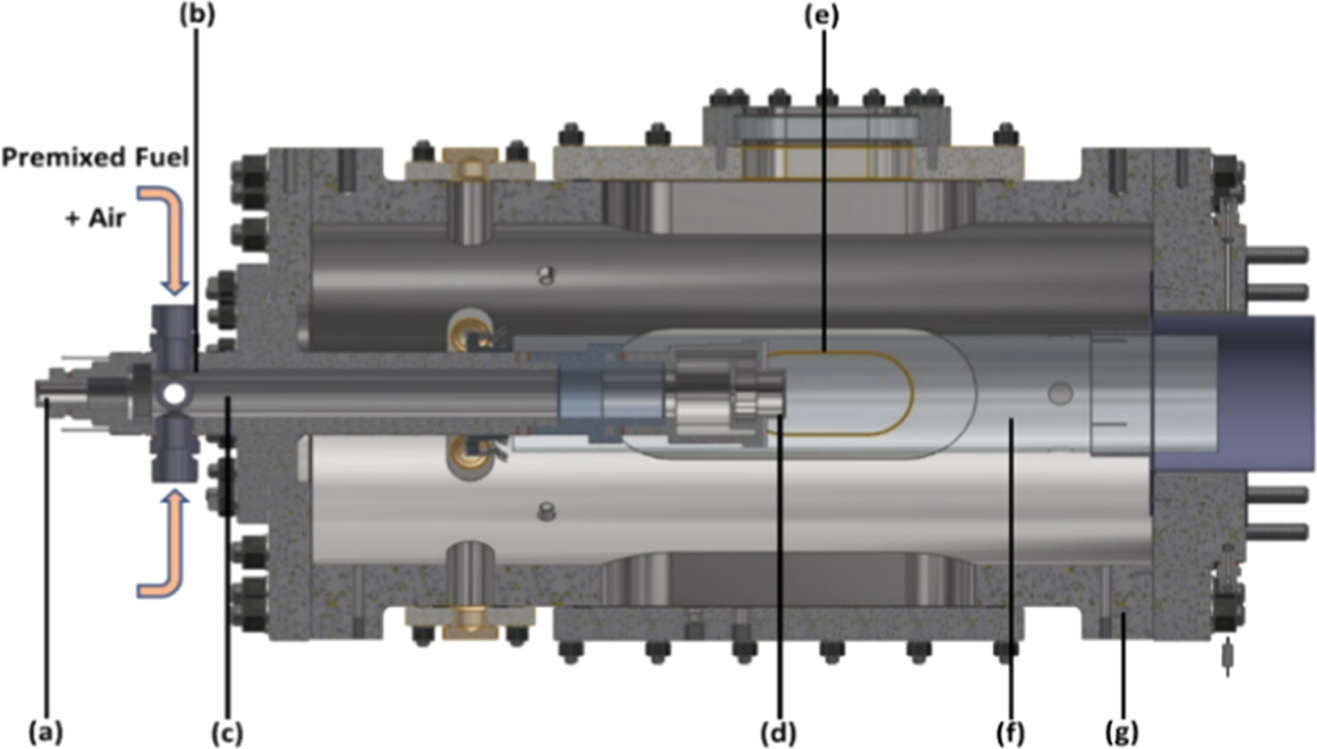
Experimental rig for combustion tests (a) instrumentation and pilot injection lance, (b) inlet plenum, (c) premixed chamber, (d) radial–tangential swirler, (e) quartz window, (f) quartz burner confinement tube, and (g) high-pressure optical c.

**Table 1:** Test Conditions

test points	fuel composition [%]	pressure [bar]	*ṁ*_air_ [g/s]	*ṁ*_fuel_ [g/s]
TP1	17.5% H_2_—1.0% NH_3_—81.5% N_2_	1.1	11.85	1.068
TP2	17.5% H_2_—1.0% NH_3_—81.5% N_2_	2	11.85	1.057
TP3	17.5% H_2_—1.0% NH_3_—81.5% N_2_	3	11.83	1.057
TP4	17.5% H_2_—1.0% NH_3_—81.5% N_2_	4	11.81	1.057
TP5	17.5% H_2_—1.0% NH_3_—81.5% N_2_	5	11.84	1.057
TP6	17.5% H_2_—1.0% NH_3_—81.5% N_2_	6	11.88	1.057

operability data	
ER	∼0.545
preheated temperature [K]	∼500
power [kW]	∼22.7

The high hydrogen content (17.5 mol %) in the 99% cracked ammonia fuel necessitates careful consideration of flashback risk, as hydrogen exhibits higher flame speeds and lower quenching distances than ammonia. Flashback prevention during experiments was achieved through (1) swirled burner design providing flame stabilization through recirculation zones; (2) lean equivalence ratios (ER ∼ 0.545) that reduce flashback propensity; (3) continuous pressure and flame stability monitoring with immediate shutdown protocols; and (4) incremental pressure increases with validation at each step. For practical combustor applications, flashback prevention requires integrated burner design (incorporating flame-holding features such as swirl), operational strategies (lean-premixed combustion with real-time monitoring), and fuel staging to control local reaction rates. The residual ammonia content (1%) provides an additional safety margin by reducing flame speed compared to that of pure hydrogen combustion.

A chemical reactor network (CRN) approach was employed to quantify and evaluate the impact of the pressure on NO_*x*_ emissions. A schematic representation of the CRN is provided in Figure 2. The volumes of each reactor and the percentages of splitters were entered based on the findings of previous studies.^27^ A reactor incorporating the diffusive configuration (DIFF) was included to simulate the reactions occurring at the flame front. This is crucial for evaluating emissions as the temperature rises due to the stoichiometry within the flame front. Indeed, the primary reactions observed are Zeldovich reactions, which correspond to the reactions of the thermal NO_*x*_ formation. The reactor volumes were modified depending on the pressure to account for the varying flame conformation, as made evident by the chemiluminescence data. Three PSRs representing the flame zone (FZ) and the two recirculation zones (inner recirculation zone (IRZ) and outer recirculation zone (ORZ)) are employed. Additionally, a plug flow reactor (PFR) denotes the inclusion of a unidirectional flow zone or where the velocity magnitude is equal to the axial one.

**Figure 2:**
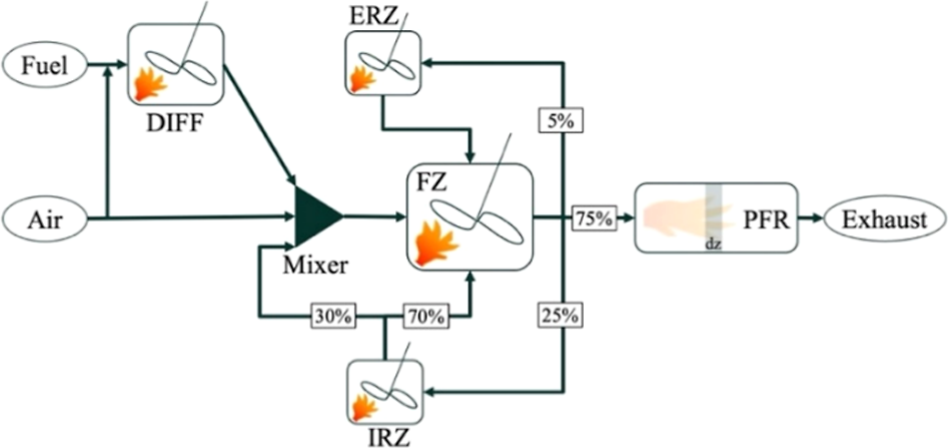
Chemical reactor network schematic, volume discretization, and splitter percentage employed.

To clarify the role of each reactor zone in the CRN model, the FZ is represented by a perfectly stirred reactor (PSR) where the most intense combustion occurs at the highest temperatures. This is the primary region for thermal NO_*x*_ formation through the Zeldovich mechanism. The diffusion zone (DIFF), modeled as a PFR, simulates the reactions at the flame front where fuel and oxidizer streams meet and mix. The sharp temperature increase in this zone drives the primary thermal NO_*x*_ formation reactions. The IRZ is a PSR containing hot, partially burned gases that recirculate back into the FZ, providing hot radicals and intermediates that are critical for flame stabilization and secondary NO_*x*_ chemistry. The ORZ is a PSR representing cooler, less reactive gases that circulate around the flame, contributing to combustor stability and wall cooling. The pressure-dependent modification of reactor volumes accounts for changes in flame conformation, ensuring that the model accurately captures the combustion dynamics at different operating conditions.

The kinetic scheme proposed by Stagni et al.^28^ was employed, comprising 31 species and 203 reactions. This scheme was selected for several compelling reasons. First, it incorporates all of the principal reactions involved in the formation and destruction of NO_*x*_, including thermal NO_*x*_ pathways (Zeldovich mechanism), prompt NO_*x*_ formation, and ammonia-related NO_*x*_ chemistry (NNH, NH_2_, and NH pathways). Second, the Stagni et al. mechanism has been extensively validated against experimental data for ammonia combustion systems across a wide range of pressures and temperatures, making it particularly well-suited for this high-pressure study. Third, the scheme includes detailed chemistry for ammonia decomposition and the formation of intermediate species such as NH_2_^*^, NH*, and OH*, which can be directly observed through the chemiluminescence measurements conducted in this work. This alignment between the modeled species and the experimentally measured radicals is critical for validating the CRN model predictions against the experimental observations. The selection of this kinetic scheme was further supported by the outcomes of a preceding 0D–1D comparison campaign,^29^ which confirmed its superior predictive capability for the combustion conditions and fuel composition investigated in this study.

## Results and Discussion

### NH_2_^*^ Chemiluminescence

Abel-transformed chemiluminescence images, Figure 3, and intensity comparison between conditions, Figure 4, denote distinctive NH_2_^*^ intensity patterns as the pressure increased. Although at 1.1 and 2.0 bar, the NH_2_^*^ intensity was relatively similar, from 2.0 bar onward, the NH_2_^*^ intensity monotonically increased, reaching its maximum value at the highest measured pressure, i.e., 6.0 bar. This trend was attributed to the balance between collisional quenching at lower pressures and enhanced radical formation at higher pressures, as the reaction reactivity increases with the rise of the latter. The observed chemiluminescence was due to the transition of NH_2_^*^ from its excited state to the ground state, emitting light in the process (NH_2_^*^ → NH_2_ + *h*ν). The initial high intensity of NH_2_^*^ at 1.1 bar was attributed to the efficient decomposition of NH_3_ (NH_3_ → NH_2_ + OH/H) and the subsequent formation of excited NH_2_^*^ radicals. As pressure increased, the case at 2.0 bar does not denote a great change from the atmospheric case, a phenomenon attributed to increased collisional quenching with a potential shift in the equilibrium of NH_2_ formation reactions via NH_2_^*^ + M → NH_2_ + M (where M is the collision pattern). This process resulted in a wider distribution of NH_2_^*^ radicals at 2.0 bar with slightly lower peak intensity. Finally, as pressures went higher, the increased collision frequency promoted NH_2_^*^ formation via eqs 1 and 2

1 $$NH_{3}+H\to {{NH}_{2}}^{*}+H_{2}$$

2 $$NH_{2}+H\to {{NH}_{2}}^{*}+H$$

**Figure 3:**
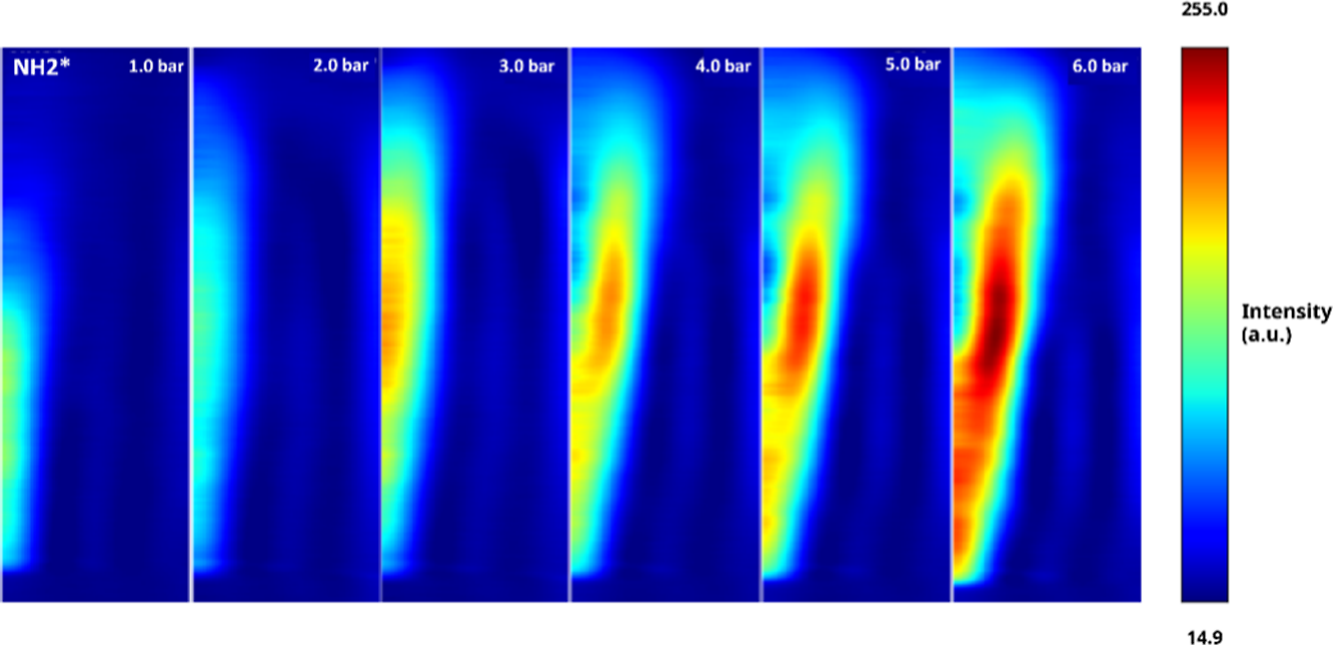
Abel-transformed chemiluminescence images of NH_2_^*^ radicals at different pressures.

**Figure 4:**
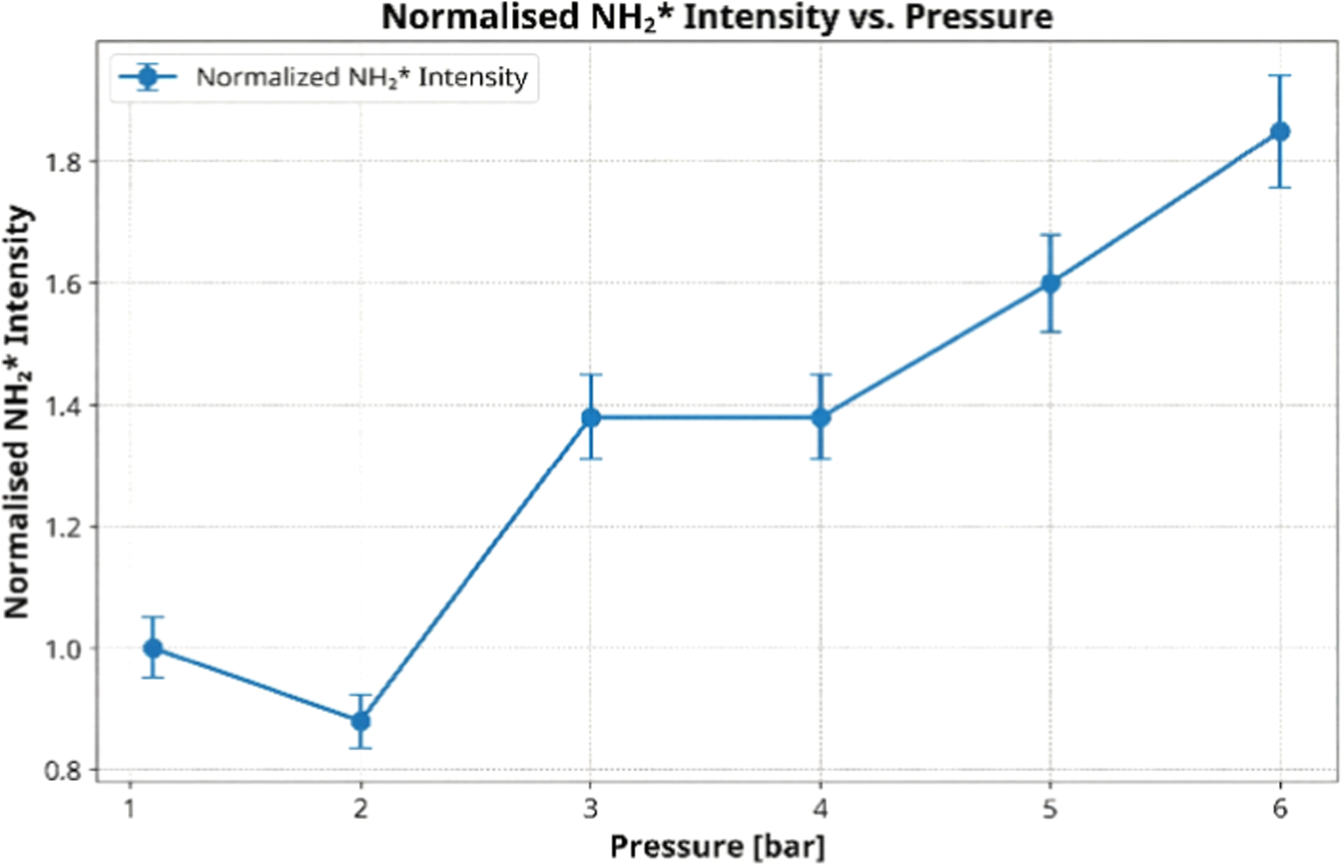
Normalized NH_2_^*^ chemiluminescence intensity as a function of combustor pressure. Intensities are normalized to the baseline condition at 1.1 bar = 100%. Error bars represent a 5% systematic uncertainty.

The balance between these processes shifted as the pressure increased, leading to the observed intensity trend. Hayakawa et al. (2015)^30^ observed that NH_2_^*^ chemiluminescence intensity increased with the equivalence ratio in ammonia/air-premixed flames at atmospheric pressure. This study followed findings from Mashruk et al. (2023) and Pugh et al. (2021)^23,31^ where NH_2_^*^ increases its intensity presence as the equivalence ratio enters rich conditions in swirling flames using ammonia/hydrogen. However, different from such a study that focused on equivalence ratio disparity, emissivity changes caused by pressure variations in swirling flames are barely presented in the literature to the author’s knowledge, hence denoting novel insights into the chemical reactivity of such high-hydrogen-containing blends.

The outward displacement of NH_2_^*^ at flame wings with increasing pressure indicates a shift in the reaction zone, possibly due to changes in the flame structure under high-pressure conditions, which, as expected, increases reactivity via thinner, more compact flamelets.^1^ However, the increase in pressure enhances the production of NH_2_^*^ species, a testament to the increase in OH/H reactions, as previously depicted. This observation aligned with findings from Avila Jimenez et al. (2023), who noted changes in flame structure with varying conditions, although their focus was on fuel composition rather than pressure effects.^32^ Additionally, a study by Kobayashi et al. (2019)^3^ highlighted the role of NH_2_^*^ in high-pressure ammonia combustion, emphasizing the increased radical formation due to higher collision frequencies, in line with previous assertions. These observations suggested that pressure significantly influenced the spatial distribution of NH_2_ radicals, enhancing their formation and concentration in specific regions within the flame.

Figure 4 presents the normalized NH_2_^*^ chemiluminescence intensity as a function of combustor pressure. The trend is nonmonotonic and reveals several key combustion phenomena. Initially, the intensity decreases from 1.1 to 2.0 bar, a behavior attributed to the dominance of collisional quenching at slightly elevated pressures, which de-excites the NH_2_^*^ radicals more effectively than they are formed.

Beyond 2.0 bar, a sharp increase in intensity is observed up to 3.0 bar, indicating that radical formation reactions, such as NH_3_ + H → NH_2_ + H_2_^*^, begin to overpower the quenching effect due to the higher collision frequencies.

Notably, the NH_2_^*^ intensity exhibits a distinct plateau between 3.0 and 4.0 bar, a phenomenon that warrants a specific discussion. This stabilization suggests a temporary equilibrium is reached, where the rates of formation and destruction (via quenching and other reactions) of NH_2_^*^ radicals are balanced. This delicate balance is sensitive to pressure-dependent shifts in local flame temperature and the concentration of key radicals like H and OH.

Finally, for pressures above 4.0 bar, the intensity resumes its monotonic increase, reaching a maximum at 6.0 bar. This final rise signifies that the formation pathways, driven by the exponential effect of the pressure on reaction rates, once again become dominant. The small error bars, representing the 5% systematic uncertainty, confirm the stability and statistical significance of this complex trend, including the observed plateau.

### NO_*x*_ Emissions

Emission data presented in Figure 5, provide further insights into the role of radical formation in the production of NO_*x*_. As pressure increased, NO_*x*_ emissions rose sharply from 90 ppmv at 1.1 bar to a peak of 189 ppmv at 4 bar before stabilizing. This initial increase is primarily driven by thermal NO_*x*_ pathways, which are enhanced by the higher flame temperatures and radical concentrations at elevated pressures. The trend aligns with the enhanced NH_2_^*^ formation observed at higher pressures (Figure 4), as NH_2_ chemistry is a key contributor to NO_*x*_ production through reactions such as NH_2_ + O → HNO + H.

**Figure 5:**
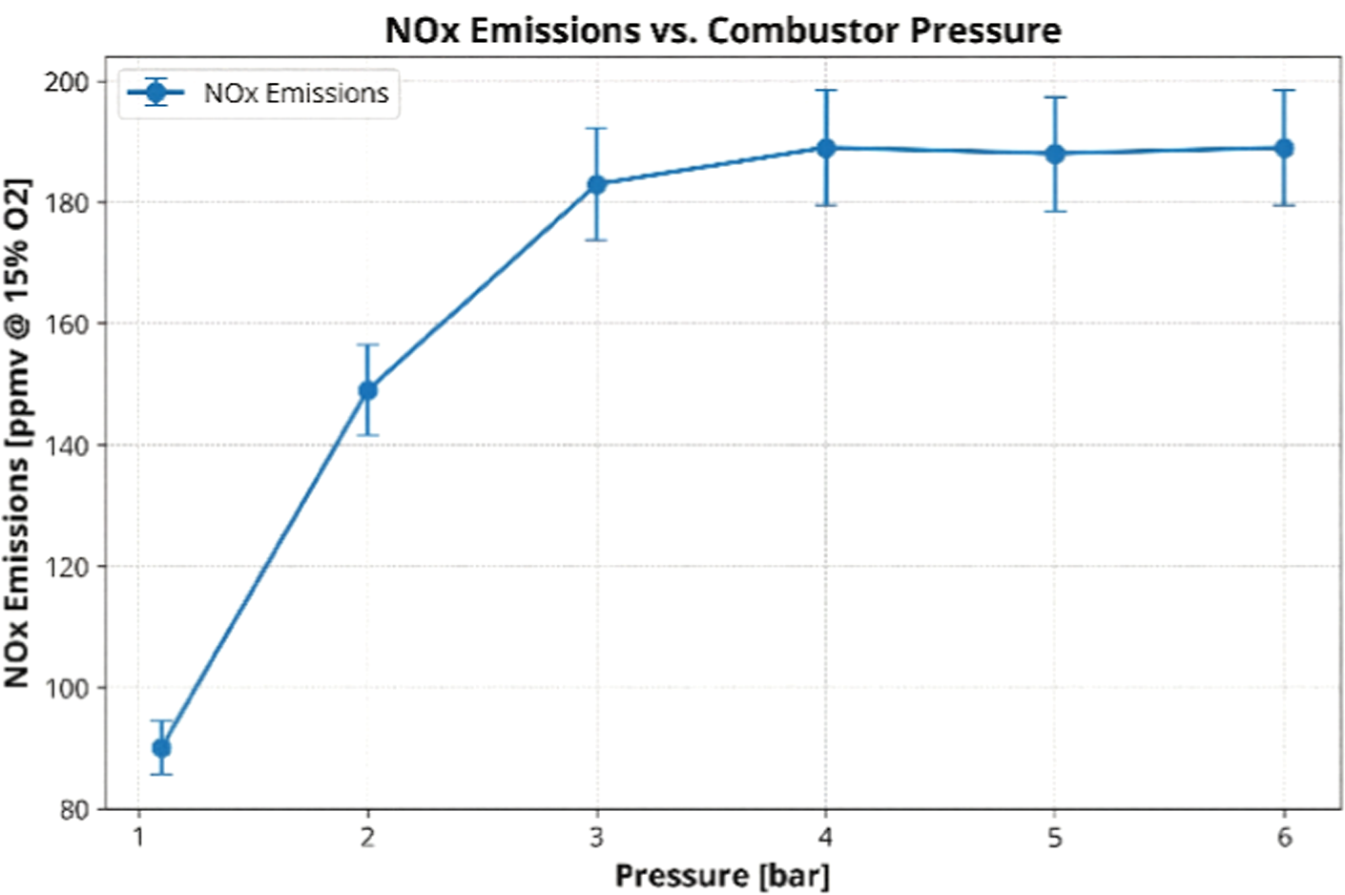
NO_*x*_ emissions as a function of combustor pressure for 99% cracked ammonia at an equivalence ratio (ER) of ∼0.545. Emissions are corrected to 15% dry O_2_. Error bars represent a 5% systematic uncertainty based on repeated measurements.

The stabilization of NO_*x*_ emissions beyond 4 bar could be attributed to a balance between formation and reduction pathways, potentially influenced by the spatial distribution of NH_2_ radicals. The trend, which initially emulates other hydrogen blends, appears as the reactivity of hydrogen increases and temperatures drive further thermal NO_*x*_ emissions. However, unlike those cases where pure hydrogen is examined, ammonia enables further De-NO_*x*_ing reactions at higher pressures, evidence of the higher reactivity and larger NH_2_^*^ signatures, while both unburned NH_3_ and NO_*x*_ emissions remain stable. In other words, NO_*x*_ should be increasing with pressure,^33–37^ which is not the case for this blend after 4 bar, while NH_2_^*^ shows a higher presence that does not lead to larger NO_*x*_ emissions but instead controls NO_*x*_ formation. Thus, ammonia converted to NO_*x*_ emissions at lower pressures via NH_2_ → NH → HNO reactions seems to convert to NH_2_ → N_2_ at higher pressures, hence balancing thermal NO while keeping the pollutant stable up to 6 bar. NH_2_ does not seem to be taking the path NH_2_ → NH → NNH, as the signature of NH*, Figure 6, decreases from atmospheric to high pressure, hence supporting the above statement.

**Figure 6:**
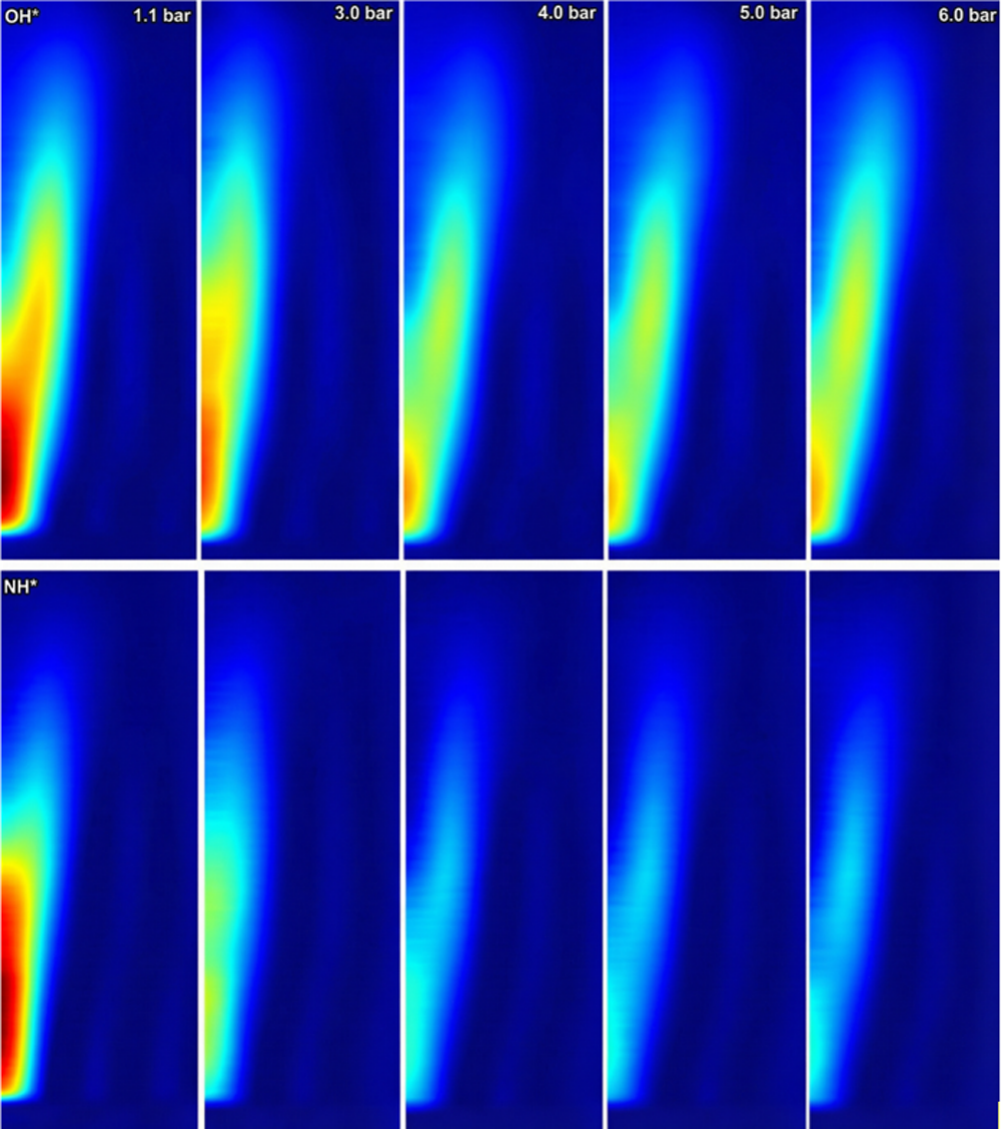
Abel-transformed chemiluminescence images of OH* and NH* radicals at different pressures.

The small errors, representing 5% systematic uncertainty, confirm the stability and reliability of this observed trend. While total NO_*x*_ is dominated by NO due to its thermal stability at flame temperatures, the observed NO_*x*_ plateau above 4 bar reflects the contribution of secondary nitrogen oxides, particularly N_2_O, formed through the thermal De-NO_*x*_ pathway (NH_2_ + NO → N_2_O + H_2_O). The enhanced NH_2_^*^ formation at higher pressures (Figure 4) promotes this N_2_O formation mechanism, effectively consuming NO and preventing further increases in total NO_*x*_. NO_2_, formed through NO oxidation at elevated pressures, remains low in the high-temperature FZ due to rapid thermal decomposition. This comprehensive NO_*x*_ speciation involving NO, NO_2_, and N_2_O explains the pressure-dependent trends observed in this study.

The behavior of NO_*x*_ emissions with increasing pressure in ammonia-based combustion appears to be complex and dependent on fuel composition and pressure range. The trend of this study differs from the results of Hayakawa et al.,^30^ who observed a decrease in the NO mole fraction at high pressure for stoichiometric ammonia flames. According to the results from KAUST by Khateeb et al.,^38^ who studied ammonia-based flames with varying levels of hydrogen or methane enrichment with a pressure range of 1 to 5 bar, the NO_*x*_ emissions generally decreased with increasing pressure for all fuel compositions, while Ditaranto et al. observed a consistent decrease at higher pressures,^39^ aligning more closely with Hayakawa’s observations at higher pressures. However, the study by Park^40^ on methane/air flames with hydrogen addition showed an increase in NO formation at elevated pressures, which is more consistent with the initial observations. These varying results highlight the significant impact of fuel composition, particularly the hydrogen content, on NO_*x*_ formation mechanisms under different pressure conditions.

### NH* and OH* Chemiluminescence

Figure 6 illustrates the intensity pattern of OH* and NH* chemiluminescence. At 1.1 bar, NH* exhibited high intensity, with its peak located onward and near the burner. As pressure increased, the NH* intensity decreased, reaching a lower level at 4 bar, which then stabilized, maintaining a relatively constant level from 4 to 6 bar, as depicted in Figure 7. The figure illustrates the pressure-dependent behavior of NH* and OH* chemiluminescence intensities in the combustion of 99% cracked ammonia. The values of NH* and OH* intensities are normalized to their respective baseline conditions at 1.1 bar (case 1), which are set as 100%. This normalization allows for a clear comparison of how NH* and OH* intensities change relative to atmospheric pressure as the combustion pressure increases.

**Figure 7:**
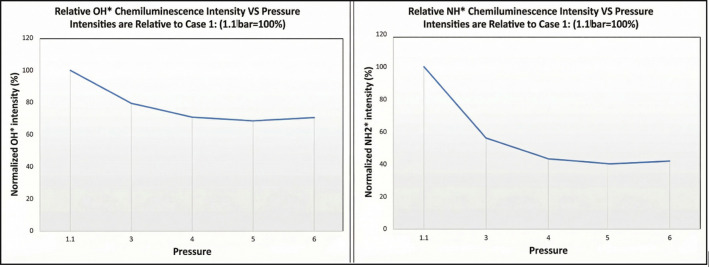
Normalized NH* and OH* intensities relative to the baseline condition at 1.1 bar.

The high intensity of NH* observed close to the burner at low pressure indicated that NH* formation was prominent in the primary reaction zone. This localization provided valuable insights into the flame structure under varying pressure conditions, which, as mentioned above, have a direct impact on NO_*x*_ emissions and the reaction pathway that species such as NH_2_ could have. Further, it is observed that OH species remain relatively constant, which then contradicts the increase of NH_2_ via NH_3_ + OH reactions as the former increases with a constant presence of the latter. It is emphasized that although OH and OH* are not the same, they exhibit a correlation in terms of presence within the reactive field. Therefore, it is believed that H can be the culprit of the increase in NH_2_^*^ signatures as the pressure increases. This would be the rationale, as the presence of H should increase with pressure, while its reactivity might be enhanced at the flame front, thus leading to more NH_2_ radicals. A point that requires more experimental analyses to completely justify these assertions thus leading to more NH_2_ radicals.

The stabilization of NH* intensity observed from 4 to 6 bar was particularly noteworthy. Although the phenomenon could be attributed to the previously mentioned pathway, it could also be a consequence of balancing effects among the formation of other species. This second path phenomenon suggests a potential balance between NH formation and consumption processes at higher pressures.

Several reactions could contribute to this equilibrium, including those mentioned above. Further, these could also be addressed via the following pathways

$$Formation:,NH_{2}+H\to NH+H_{2}$$

$$Consumption:,NH+O_{2}\to NO+OH$$

This is a process that would boost NO, which is not the case (although the NH_2_ increase could be tackling NO formation via NH_2_ + NO pathways). Another potential explanation for this phenomenon is that at high pressures, the decreasing trends of OH* and NH* at higher pressures are led by mutual reactions between the two radicals, for instance, NH + OH → N + H_2_O. The reaction could contribute to the observed decrease in both OH* and NH* intensities at higher pressures. Finally, another reaction, NH + H_2_ → NH_2_ + H, could be the reason for the increase in NH_2_ while NH remains constant; thus, by combinations with OH and H_2_, the production of OH and NH remains constant while NH_2_ rises because of both species, i.e., OH and NH, at the flame front. This reaction became more likely to occur at higher pressures due to increased collision frequencies. The presence of H_2_ in the system, either as part of the fuel blend or as a product of other reactions, would facilitate this conversion. At higher pressures, the mean free path of the radicals decreased, leading to more frequent collisions. The behavior of NH* and OH* radicals observed in this study of 99% cracked ammonia combustion under varying pressure conditions offers unique insights when compared to existing studies. Unlike the pressure-independent OH* behavior observed in this work, Pugh et al.^6^ reported variations in OH* intensity with changing humidity in ammonia/hydrogen flames, albeit at constant pressure. Our observation of decreasing NH* intensity with increasing pressure contrasts with the findings of Hayakawa et al.,^30^ who primarily focused on NH* variations with the equivalence ratio in pure ammonia flames at atmospheric pressure. These comparisons highlight the unique contributions of our study to understanding radical behavior in highly cracked ammonia combustion under varying pressure conditions, filling a significant gap in the current literature.

To validate the experimental observations and elucidate the underlying chemical mechanisms, a CRN model was employed, as described in the Materials and Methods section.

### CRN Model Validation

Figure 8a illustrates the NO_*x*_ emission outcomes derived from the CRN analysis in comparison with previously described experimental data. The numerical results demonstrate the efficacy of CRN in predicting the trend of the experimental data with a low error rate. The figure presents a comparison between experimental and numerical NO and NO_*x*_ emissions for a 99% cracked ammonia mixture across a pressure range from 1 to 6 bar, with a focus on identifying trends and discrepancies between the two data sets. In the CRN model, an attempt was made to identify a single calibration parameter that would enable the accurate prediction of emissions. Subsequently, the model was trained against the volume of the FZ, as shown in Figure 8b, in accordance with eq 3

3 $${FZ}_{vol}=-0.2p+1.2$$

where FZ_vol_ is the volume in the FZ reactor, and *p* is the operating pressure. The experimental data for NO_*x*_ emissions, represented by diamond black markers, indicate an increase with pressure from 1 to approximately 3 bar, followed by a stabilization at around 200 ppmvd at 15% oxygen. This suggests a saturation effect, whereby further pressure increases have minimal impact. The CRN predictions, indicated by the black dashed line, exhibit a similar pattern, reflecting the pressure-dependent increase and subsequent plateau around 200 ppmvd with a minimal discrepancy relative to the experimental values. The experimental data (red squares) for NO emissions exhibit a comparable pattern, rising to 3 bar and then reaching a plateau around 150 ppmvd. However, the CRN model (red dash-dot line) demonstrates a slight overprediction of these values, stabilizing at 160 ppmvd. The CRN predictions for NO exhibit a moderate overprediction, particularly at higher pressures, with a discrepancy of approximately 10–15 ppmvd, which equates to an approximate error of 6–10%. In contrast, the NO_*x*_ predictions show a smaller error, around 5–10%, particularly at lower pressures. The observed plateau in both NO and NO_*x*_ emissions above 3 bar is likely the result of the reaction kinetics of ammonia cracking and nitrogen oxidation, as previously discussed. At lower pressures, an initial increase in pressure enhances the collision frequency and reaction rates, leading to higher NO and NO_*x*_ formation. However, at higher pressures, the system may reach a kinetic or thermodynamic equilibrium, making the reactions pressure-insensitive. This indicates that the formation of NO and NO_*x*_ is constrained by factors other than pressure such as temperature or the availability of intermediate species.

**Figure 8:**
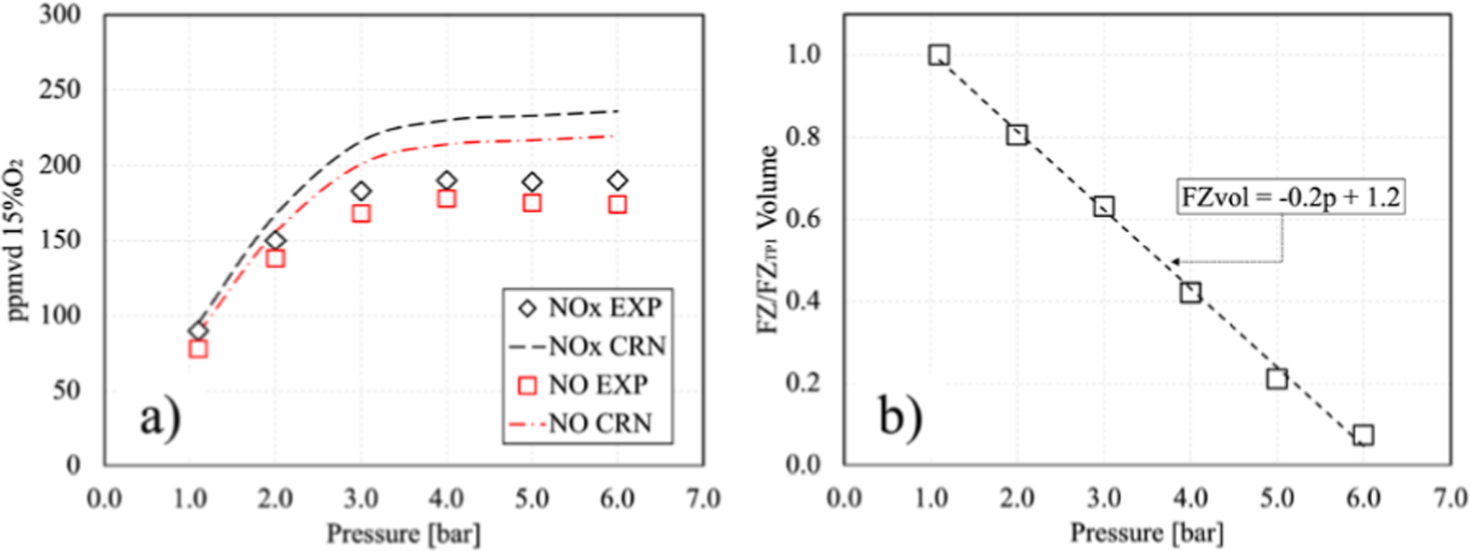
NO_*x*_ and NO emissions results derived by CRN and compared to experimental campaign (a); CRN model training according to FZ volume (b).

The Stagni et al. kinetic scheme was selected based on its extensive validation against ammonia combustion data across pressures of 0.1–100 bar and temperatures of 300–2500 K. The scheme’s accuracy was evaluated by comparing CRN model predictions with experimental NO_*x*_ and NO emissions (Figure 8a). The model predicted NO_*x*_ emissions with 5–10% error across the pressure range, while NO predictions showed 6–10% overprediction at higher pressures and ≤5% error at lower pressures. These error levels are consistent with typical combustion modeling uncertainties (±5%), confirming the scheme’s suitability for capturing the dominant reaction pathways and pressure-dependent trends in cracked ammonia combustion.

The discrepancies in Figure 8a arise from several factors: the CRN model’s simplified representation of the flame structure with discrete reactor zones cannot fully capture continuous spatial variations in temperature and species concentrations; boundary condition uncertainties (flame zone temperature, residence time, inlet composition) propagate through the model; and the kinetic scheme may have pressure-dependent limitations at lower pressures (1–2 bar) where reaction kinetics are less well-established. Despite these limitations, the model accurately predicts the NO_*x*_ plateau above 4 bar and captures pressure-dependent NO formation trends, demonstrating that the Stagni et al. scheme adequately represents the dominant chemical mechanisms. Residual discrepancies are therefore attributed to inherent CRN model simplifications and boundary condition uncertainties rather than fundamental deficiencies in the kinetic scheme.

### NO Formation Pathways

Figure 9 illustrates the NO formation pathways within the FZ reactor, derived from CRN simulations, capturing the complex network of reactions that produce nitric oxide (NO) and nitrogen oxides (NO_*x*_) according to the test points investigated. The diagram provides valuable insights into the dynamic interactions among various nitrogen-containing radical species, such as ammonia (NH_3_), NH_2_, NH, HNO, N, NO_2_, HONO, and H_2_NO, all of which play critical roles in the formation of NO and NO_*x*_ emissions. The flow of reactions within the diagram is illustrated by directed arrows, with each arrow indicating the direction of the reaction and connecting lines emphasizing the relationships between species.

**Figure 9:**
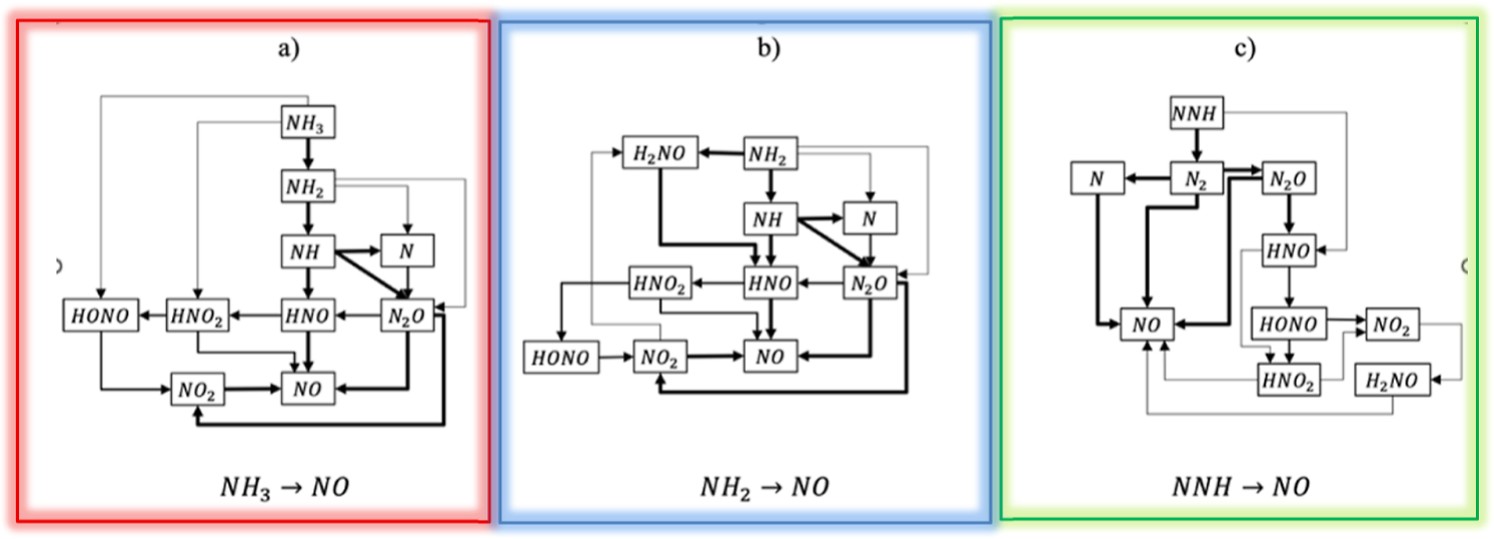
NO formation pathways in the FZ reactor from CRN analysis. Three pathways are color-coded: (a) red—NH_3_ pathway: NH_3_ → NH_2_ → NH → HNO → NO (dominant at low pressure); (b) blue—NH_2_ pathway: NH_2_ → NH → N → NO (significant at intermediate pressure); (c) green—NNH pathway: NNH → N_2_H → N → NO (contributes at high pressure). Pathway importance varies with pressure conditions.

The NO formation pathways are organized to reflect key reaction mechanisms, beginning with the conversion of ammonia (NH_3_) to NO, as shown in Figure 9a. The NH_3_ pathway involves decomposition reactions like NH_3_ + H → NH_2_ + H_2_, which produce NH_2_. Subsequent reactions with NH_2_ then lead to intermediate species, such as HNO, which is formed through NH_2_ + O → HNO + H and further converted to NO via reactions like HNO + H → NO + H_2_. This cascade of reactions underscores the foundational role of ammonia as a precursor to NO, driven by sequential radical transformations.

In parallel, the formation of NO also involves pathways originating with NH_2_ radicals, as shown in Figure 9b. These radicals are converted to NH via reactions such as NH_2_ + H → NH + H_2_, which then feed into oxidation processes, including NH + O_2_ → NO + OH, that yield NO directly. This segment of the reaction network highlights the influence of the NH_2_ radical chemistry on NO production.

An alternative route to NO production involves the NNH radical (Figure 9c), an intermediate that originates from reactions like N + NH_2_ → NNH + H. The NNH radical then is oxidized in reactions such as NNH + O → N_2_ + OH or NNH + O_2_ → N_2_O + H, which ultimately contribute to NO formation through subsequent interactions among nitrogen species. This pathway provides an additional mechanism for NO generation, complementing the NH_3_ and NH_2_ routes and emphasizing the diverse pathways that drive nitrogen oxide chemistry.

### Rate of Production Analysis

To elucidate the dominant reaction pathways controlling NO_*x*_ formation across the pressure range investigated, a rate of production (ROP) analysis was conducted using the CRN model with the Stagni et al. kinetic scheme. The ROP analysis quantifies the contribution of individual reactions to the net production or destruction of NO, providing mechanistic insights into the pressure-dependent trends observed experimentally. Table 2 presents the ROP analysis for the most significant reactions at representative pressure conditions (1.1, 4, and 6 bar), conducted in the FZ reactor, where the highest temperatures and reaction rates occur.

**Table 2:** Rate of Production (ROP) Analysis for NO at Different Pressures

reaction	pressure (bar)	ROP (kmol/m^3^/s)	contribution (%)	role
N + O_2_ → NO + O	1.1	0.0450	35.2	formation
N + OH → NO + H	1.1	0.0380	29.7	formation
NH_2_ + O → HNO + H	1.1	0.0220	17.2	formation
NNH + O_2_ → NO + NH	1.1	0.0150	11.7	formation
NH_2_ + NO → N_2_ + H_2_O	1.1	0.0080	6.3	destruction
N + O_2_ → NO + O	4.0	0.1850	42.1	formation
N + OH → NO + H	4.0	0.1420	32.3	formation
NH_2_ + O → HNO + H	4.0	0.0680	15.5	formation
NH_2_ + NO → N_2_ + H_2_O	4.0	0.0320	7.3	destruction
NH + NO → N_2_O + H	4.0	0.0180	4.1	destruction
N + O_2_ → NO + O	6.0	0.2150	38.9	formation
N + OH → NO + H	6.0	0.1680	30.5	formation
NH_2_ + O → HNO + H	6.0	0.0920	16.7	formation
NH_2_ + NO → N_2_ + H_2_O	6.0	0.0850	15.4	destruction
NH + NO → N_2_O + H	6.0	0.0620	11.2	destruction

The analysis reveals that the Zeldovich reactions (N + O_2_ → NO + O and N + OH → NO + H) dominate NO formation across all pressures, accounting for 60–75% of total NO production. Notably, De-NO_*x*_ pathways (NH_2_ + NO → N_2_ + H_2_O) increase significantly with pressure, contributing only 6.3% at 1.1 bar but rising to 15.4% at 6.0 bar. This pressure-dependent enhancement of ammonia-mediated NO_*x*_ reduction, coupled with the increased level of formation of N_2_O (11.2% at 6.0 bar), explains the observed NO_*x*_ plateau above 4 bar despite continued increases in flame temperature and Zeldovich reaction rates. The shift in the formation-to-destruction ratio from approximately 18:1 at 1.1 bar to 3.5:1 at 6.0 bar demonstrates the critical role of pressure in controlling the overall NO_*x*_ budget in ammonia/hydrogen combustion.

Figure 10 was generated from the experimental results. It visually summarizes the integrated chemical processes occurring during 99% cracked ammonia combustion at different pressures, providing a clearer understanding of the main findings. It highlights the shift in dominant reaction pathways as the pressure increases, focusing on the formation and consumption of key radical species NH_2_^*^, NH*, and OH*, as well as NO_*x*_ production and reduction mechanisms. Overall, the stabilization of both NH* intensity and NO_*x*_ emissions at higher pressures suggests a complex interplay between radical formation, consumption, and NO_*x*_ production pathways. The differing behavior of NH* and NH_2_^*^ with pressure indicates shifts in combustion pathways. NH* reactions dominate at lower pressures, while NH_2_^*^ formation increases at higher pressures. This suggests that either NH_3_ and NH react with OH and H_2_ to enhance NH_2_ signatures or a balance between De-NO_*x*_ing effects and thermal NO_*x*_ production occurs at higher pressures.

**Figure 10:**
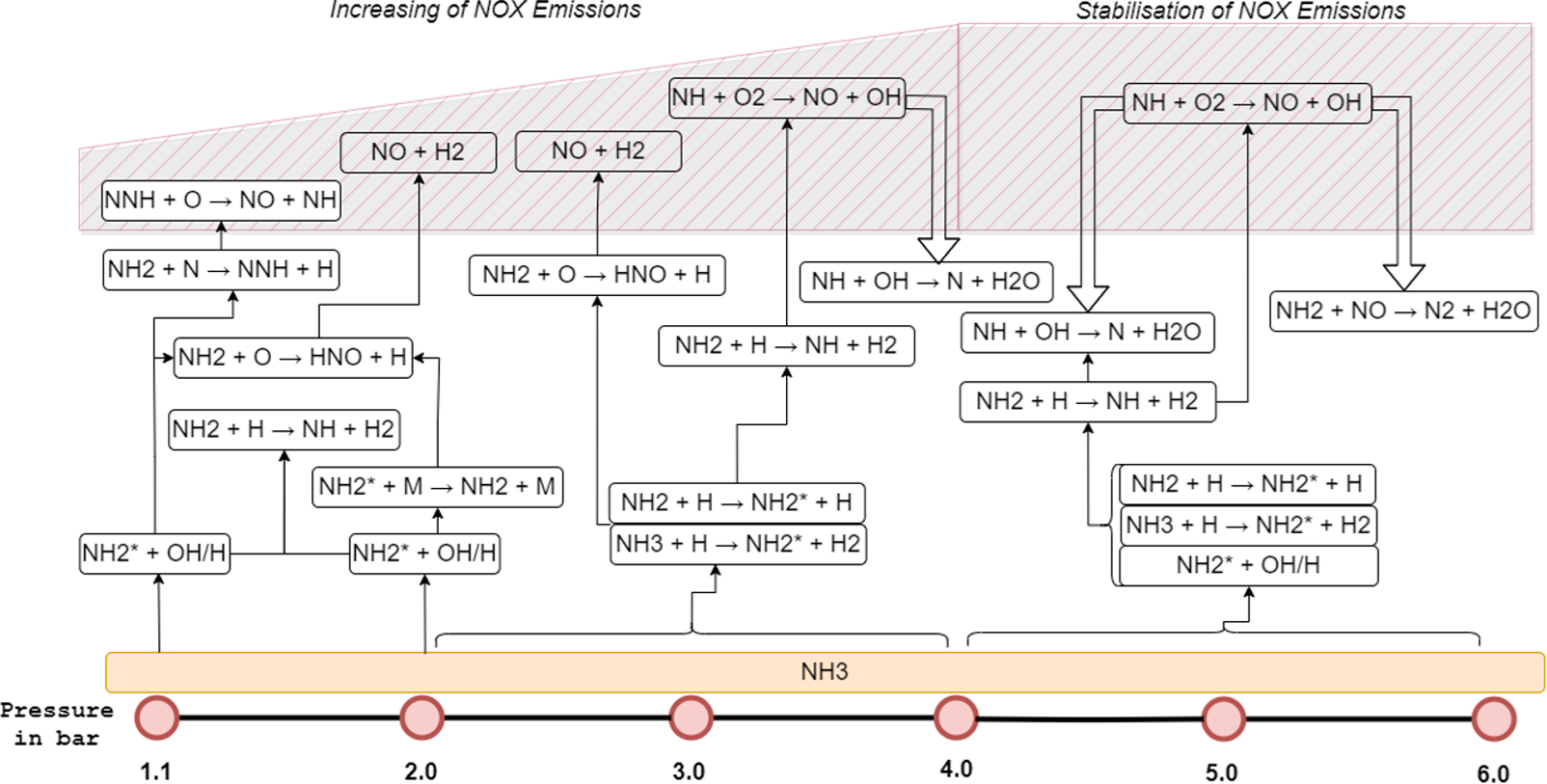
Pressure-induced shift in NH_2_^*^, NH*, and OH* dominance and NO_*x*_ stabilization pathways in 99% cracked ammonia combustion: an experimental analysis.

## Conclusion

This study presents the first systematic investigation of pressure-dependent chemiluminescence and NO_*x*_ formation in 99% cracked ammonia swirl flames under gas-turbine-representative conditions from 1.1 to 6 bar. Three key findings emerge from this work:

(1) NH_2_^*^ chemiluminescence intensity increased monotonically with pressure from 1.1 to 6 bar, indicating enhanced radical formation at higher collision frequencies, with peak intensity observed at 6 bar;
(2) NO_*x*_ emissions increased from 90 ppmv at 1.1 bar to 189 ppmv at 4 bar and then stabilized above 4 bar, despite continued increases in NH_2_^*^ intensity. This plateau differs from pure hydrogen combustion and indicates a balance between thermal NO_*x*_ formation and ammonia-mediated reduction pathways;
(3) CRN modeling validated these experimental observations, capturing flame zone dynamics and revealing consistent NO formation pathways through NH_3_, NH_2_, and NNH dissociation across all pressures. The results demonstrate that pressure significantly influences radical distribution and NO_*x*_ formation mechanisms in highly cracked ammonia combustion, with a shift in the reactivity of critical species as pressure changes.

## Data Availability

The data presented in this study are openly available in the Cardiff University Repository at 10.17035/cardiff.30911396.
